# Antibacterial Activity of Isobavachalcone (IBC) Is Associated with Membrane Disruption

**DOI:** 10.3390/membranes12030269

**Published:** 2022-02-25

**Authors:** Leticia Ribeiro de Assis, Reinaldo dos Santos Theodoro, Maria Beatriz Silva Costa, Julyanna Andrade Silva Nascentes, Miguel Divino da Rocha, Meliza Arantes de Souza Bessa, Ralciane de Paula Menezes, Guilherme Dilarri, Giovane Böerner Hypolito, Vanessa Rodrigues dos Santos, Cristiane Duque, Henrique Ferreira, Carlos Henrique Gomes Martins, Luis Octavio Regasini

**Affiliations:** 1 Department of Chemistry and Environmental Sciences, Institute of Biosciences, Humanities and Exact Sciences, São Paulo State University (Unesp), São José do Rio Preto 15054-000, SP, Brazil; leticia.assis@unesp.br (L.R.d.A.); reinaldo.theodoro@unesp.br (R.d.S.T.); mb.costa@unesp.br (M.B.S.C.); jullyana.as.nascentes@unesp.br (J.A.S.N.); miguelquimic@gmail.com (M.D.d.R.); 2 Department of Microbiology, Institute of Biomedical Sciences, Federal University of Uberlândia (UFU), Umuarama 38405-320, MG, Brazil; melizaarantes@gmail.com (M.A.d.S.B.); ralciane@ufu.br (R.d.P.M.); carlos.martins2@ufu.br (C.H.G.M.); 3 Department of Biochemistry and Microbiology, Institute of Biosciences, São Paulo State University (Unesp), Rio Claro 130506-900, SP, Brazil; gui_dila@hotmail.com (G.D.); giovane.boerner@unesp.br (G.B.H.); henfer72@gmail.com (H.F.); 4 Department of Preventive and Restorative Dentistry, School of Dentistry, São Paulo State University (Unesp), Araçatuba 16015-050, SP, Brazil; vanessarodrigues_22@hotmail.com (V.R.d.S.); cristianeduque@yahoo.com.br (C.D.)

**Keywords:** chalcone, membrane, natural product, antibacterial, biofilm

## Abstract

Isobavachalcone (**IBC**) is a natural prenylated chalcone with a broad spectrum of pharmacological properties. In this work, we newly synthesized and investigated the antibacterial activity of **IBC** against Gram-positive, Gram-negative and mycobacterial species. **IBC** was active against Gram-positive bacteria, mainly against Methicillin-Susceptible *Staphylococcus aureus* (MSSA) and Methicillin-Resistant *Staphylococcus aureus* (MRSA), with minimum inhibitory concentration (MIC) values of 1.56 and 3.12 µg/mL, respectively. On the other hand, **IBC** was not able to act against Gram-negative species (MIC > 400 µg/mL). **IBC** displayed activity against mycobacterial species (MIC = 64 µg/mL), including *Mycobacterium tuberculosis*, *Mycobacterium avium* and *Mycobacterium kansasii*. **IBC** was able to inhibit more than 50% of MSSA and MRSA biofilm formation at 0.78 µg/mL. Its antibiofilm activity was similar to vancomycin, which was active at 0.74 µg/mL. In order to study the mechanism of the action by fluorescence microscopy, the propidium iodide (PI) and SYTO9 fluorophores indicated that **IBC** disrupted the membrane of *Bacillus subtilis*. Toxicity assays using human keratinocytes (HaCaT cell line) showed that **IBC** did not have the capacity to reduce the cell viability. These results suggested that **IBC** is a promising antibacterial agent with an elucidated mode of action and potential applications as an antibacterial drug and a medical device coating.

## 1. Introduction

Approximately 1 million people died due to antibiotic-resistant infections between 2014 and 2016 around the world, and there is a projection that multidrug resistance will lead 300 million people to premature deaths until 2050 [1,2]. The fast and global spread of multidrug-resistant bacteria has been recognized as a great challenge to be overcome in the 21st century. Thus, strong efforts are necessary to investigate new antibacterial drugs, which must be active against resistant pathogens to current anti-infective therapy [2].

**Figure 1:**
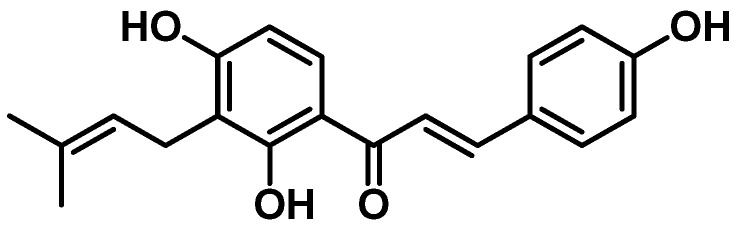
Structure of Isobavachalcone (**IBC**).

Natural products from plants and microorganisms are sources of new antibacterial compounds [3]. Among the 162 antibacterial agents approved by FDA between 1981 and 2019, about 55% are natural products and their derivatives, highlighting their relevance to modern drug discovery [3,4]. Among the promising natural products, isobavachalcone (**IBC**) is a prenylated chalcone isolated from plants of the Fabaceae, Clusiaceae, Moraceae, Schisandraceae and Apiaceae families (Figure 1) [5,6]. Moreover, the concise synthesis of **IBC** has been described by several groups [7,8,9,10,11]. **IBC** is a privileged compound due to its extensive pharmacological properties, including antibacterial [6], anti-cancer [12], antifungal [13], antioxidant [14], neuroprotective [15] and anti-inflammatory [16] properties.

As part of our continuing search for novel antibacterial drugs that act on bacterial membranes, we newly synthesized **IBC** and evaluated its antibacterial activity against Gram-positive, Gram-negative and *Mycobacterium* planktonic cells. Additionally, we investigated the antibacterial activity of **IBC** against biofilms of Methicillin-susceptible *Staphylococcus aureus* (MSSA) and Methicillin-resistant *Staphylococcus aureus* (MRSA). **IBC** was tested regarding its effects on the membrane of *Bacillus subtilis*, as well as its toxicity toward human skin cells.

## 2. Materials and Methods

### 2.1. Isobavachalcone (IBC)

The synthesis **of IBC** was performed according to Sugamoto and collaborators, using six steps [8]. The structure of **IBC** was confirmed by ^1^H and ^13^C nuclear magnetic resonance (NMR) and mass spectrometry (MS) data analyses. The purity of **IBC** was determined by high-performance liquid chromatography with a photodiode array detector (HPLC-PAD). Detailed synthetic experimental procedures, NMR spectra, mass spectrum and the HPLC-PAD chromatogram are presented in the Supplementary Material.

### 2.2. Antibacterial and Antimycobacterial Assays

The strains were directly purchased from the American Type Culture Collection (ATCC) and were maintained in the culture collection of the Laboratory of Antimicrobial Testing of the Federal University of Uberlândia state of Minas Gerais, Brazil.

The antibacterial activity was determined against Methicillin-susceptible *Staphylococcus aureus* (ATCC 6538), Methicilin-resistant *Staphylococcus aureus* (ATCC BAA44), *Streptococcus pneumoniae* (ATCC 6305), *Streptococcus sanguinis* (ATCC 10556), *Streptococcus sobrinus* (ATCC 33478), *Streptococcus mutans* (ATCC 25175), *Klebsiella pneumoniae* (ATCC 10031) and *Pseudomonas aeruginosa* (ATCC 15442). The antimycobacterial activity was determined against *Mycobacterium tuberculosis* H37Rv (ATCC 27294), *Mycobacterium avium* (ATCC 25291) and *Mycobacterium kansasii* (ATCC 12478).

The minimal inhibitory concentration (MIC) against Gram-positive and Gram-negative bacterial species was determined in triplicate using the broth microdilution method in 96-well microplates as previously reported, using resazurin as a colorimetric indicator of cell viability [17]. **IBC** was dissolved in DMSO at 1 mg/mL, followed by dilution in brain heart infusion to achieve the final concentrations ranging from 400.0 to 0.195 μg/mL. The final DMSO content was 5% (*v*/*v*), and this solution was used as the negative control. The inoculum was adjusted for each microorganism to reach a cell suspension of 5.0 × 10^5^ colony-forming units per mL (CFU/mL), as preconized by the Clinical and Laboratory Standards Institute, with modifications in the culture medium [18]. Growth control (inoculated well) and sterility control (non-inoculated well free of antimicrobial agent) were also included. Tetracycline and chlorhexidine were used as positive controls and were tested in concentrations ranging from 0.0115 μg/mL to 5.9 μg/mL and 0.115 to 59 μg/mL, respectively. The 96-well microplates were sealed with plastic film and incubated at 37 °C for 24 h. After this period, 30 μL of aqueous solution of resazurin (0.02%) was added to the microplates and was incubated for 15 min at 37 °C. MIC value was defined as the lowest concentration able to inhibit the microorganism growth indicated by the color change of resazurin from blue to pink.

MIC values of **IBC** against mycobacteria species were determined according to Palomino and collaborators protocol, with slight modifications [19,20]. A stock solution of **IBC** was prepared in DMSO and diluted in Middlebrook 7H9 broth to achieve final concentrations ranging from 7.8 to 1000 µg/mL. Isoniazid was dissolved in DMSO and used as positive control in concentrations ranging from 0.015 to 1.0 µg/mL. The inoculum was prepared by introducing a range of colonies grown in Ogawa-Kudoh in a tube containing glass beads with 500 μL of sterile water. An aliquot of 200 μL was transferred to a tube containing 2 mL of 7H9 broth, incubated at 37 °C for 7 days and compared with McFarland scale 1 (3.0 × 10^8^ cells/mL). Inoculum was suspended in 96-well plates at a 1:25 ratio with 7H9 broth. The growth controls (without antibiotic) and sterility controls (without inoculation) were also included. The 96-well plates were incubated at 37 °C for 7 days. After this period, 30 µL resazurin 0.02% aqueous solution was added to each plate well. The MIC value was defined as the lowest drug concentration able to inhibit the mycobacterial growth, which was expressed in µg/mL. The assay was conducted in triplicate.

### 2.3. Checkerboard Assay

The combination effect of **IBC** with vancomycin was evaluated against MSSA and MRSA using microdilution broth checkerboard assay according to White and collaborators, adapted from the standard procedure established by CLSI [18,21]. The fractional inhibitory concentration (FIC) of the combination between **IBC** and vancomycin was determined using Mueller–Hinton broth culture medium into 96-well plates, with a final inoculum suspension of 5.0 × 10^5^ CFU/mL. The plates were incubated at 37 °C for 24 h. After incubation, 0.02% aqueous resazurin solution was added to the wells. Fractional inhibitory concentration index (FICI) was calculated using the Equation (1):

ΣFICI = FICA + FICB(1)

where the FIC is the ratio between the MIC of the drug in combination with the MIC alone. The combination was classified as synergistic (FICI ≤ 0.5), additive (1> FICI > 0.5), indifferent (4 > FICI > 1) and antagonistic (FICI ≥ 4) [21]. The assays were performed in triplicate on independent experiments.

### 2.4. Antibiofilm Assay

The inhibition of **IBC** against MSSA and MRSA biofilm formation was evaluated using broth microdilution methodology proposed by CLSI (2012) [18]. The minimum biofilm inhibitory concentration (MBIC) was established as the concentration of **IBC** able to inhibit 50% or more of biofilm formation [22]. The MBIC values were determined using two protocols, including biomass assessment by optical density (OD) reading and determination of viable biofilm cells by counting colony-forming units per milliliter (CFU/mL). Two microplates were used for each protocol. Assays were performed in triplicate in three independent experiments. The inoculum concentration and optimal incubation time for this assay were determined by standardizing biofilm formation (data not shown).

Briefly, in 96-well flat-bottom microplates containing brain heart infusion (BHI) broth supplemented with 2% glucose, serial dilutions of the samples were made from the stock solution (1600 µg/mL), obtaining a final concentration between 0.195 to 400 µg/mL. From a 24 h culture on BHI agar plates, the inoculum of *S. aureus* was prepared in BHI broth supplemented with 2% glucose, with equivalent turbidity on a spectrophotometer operating at 625 nm, to match 0.5 in the McFarland scale (1.5 × 10^8^ CFU/mL). The bacterial suspensions were diluted to the final concentration of 1.0 × 10^6^ CFU/mL. The microplates were incubated at 37 °C for 24 h. Subsequently, the contents of the wells were aspirated, and non-adhered cells were removed by washing with a phosphate-buffered saline (PBS) buffer (pH = 7.2). The biofilm formed was fixed with methanol for 15 min, dried at room temperature and stained with a crystal violet solution (0.2%) for 20 min. After removing the crystal and washing the wells with the PBS buffer, 33% acetic acid was added for 30 min to solubilize the crystal retained in the biofilm. The absorbance of the wells was determined in a spectrophotometer at 595 nm. The determination of MBIC was performed using Equation (2) [22,23].

MBIC = (A_595_ of the test ÷ A_595_ of the untreated control) × 100 (2)

For the determination of viable cells, plates were prepared following the same methodology described above. After the incubation period, the contents of each well were aspirated and washed with PBS buffer to remove non-adhered cells. Then, 200 µL of BHI broth with 2% glucose was added to the wells and the microplate was subjected to an ultrasound bath for 15 min. The content of the wells was homogenized, and decimal dilutions were performed (100 to 10^−7^). After that, 50 µL aliquots of each dilution were plated on BHI agar plates and incubated at 37 °C for 24 h. Finally, the colonies were counted, and the results were expressed in log_10_ scale (CFU/mL).

Vancomycin was used as positive control and was tested in concentrations ranging from 0.0115 to 5.9 µg/mL. Bacterial cells were evaluated in the absence of the antibacterial compounds and were used as negative control. Antibiofilm assay data were analyzed by nonparametric Kruskal–Wallis one-way analysis of variance (ANOVA) with a Steel–Dwass–Critchlow–Fligner pairwise comparison test. Results were considered statistically significant with *p* < 0.05.

### 2.5. Membrane Disruption Assay

For the membrane permeabilization assay, *Bacillus subtilis* strain 168 was kindly donated by Dr. Frederico Gueiros-Filho, Department of Biochemistry, Institute of Chemistry, São Paulo University.

*B*. *subtilis* was cultivated in LB/LB-agar at 30 °C, with stirring at 200 rpm for liquid medium. A stock solution of **IBC** at 10 mg/mL was diluted into the wells of a 96-multiwell plate to furnish the final concentrations ranging from 100 to 0.781 µg/mL, in total volumes of 100 µL/well (NYG medium) [24,25]. The bacteria were inoculated at 1.0 × 10^5^ cells per 100 µL of LB medium per well. Plates were incubated for 12 h at 30 °C. 15 µL of 0.1 mg/mL resazurin Sigma-Aldrich (Taufkirchen, Germany) was added into each well, followed by a 2 h incubation at 30 °C. The post-reaction plates were evaluated through excitation and emission wavelengths at 530 and 590 nm, respectively, using the Fluorescence Synergy H1N1. The data obtained were used to plot the concentration of the compound versus cell growth inhibition, and through a polynomial curve regression, it was possible to determine the percentages able to inhibit cellular metabolism [26].

The minimum bactericidal concentration (MBC) was established by inoculating the contents of each REMA well into a 15 cm Petri dish with solid LB medium before the resazurin addition. The microbial transfer was aided by a stamping replicator fit for a 96-well microtiter plate. The cells were incubated in triplicates at 30 °C for 24 h to quantify their growth.

Cells of *B*. *subtilis* were exposed to the **IBC** at its MBC. In 1 microcentrifuge tubes, 100 μL of 1.0 × 10^5^ cells were used per treatment. After 15 min, 900 μL of saline solution (0.85%) were added to each tube to dilute the compound and stop the contact reaction. For the membrane integrity analyses, cells were stained using the Live/Dead BacLight kit following the instructions of the manufacturer. Cells treated with 1% DMSO and nisin (5 µg/mL) were used as negative control and positive control, respectively [26]. Cells were immobilized on agarose-covered slides before the microscope observations with Olympus BX-61 microscope, equipped with a monochromatic OrcaFlash-2.8 camera. Image acquisition and processing were performed with the software CellSens version 11 (Olympus). Data analyses were conducted with a minimum of 100 cells per treatment [27].

### 2.6. Cytotoxicity Assay

Human keratinocytes cells (HaCaT) were cultivated in Dulbecco’s Modified Eagle Medium (DMEM; Gibco, Grand Island, NY, USA) supplemented with 10% fetal bovine serum (FBS), penicillin (100 IU/mL), streptomycin (100 μg/mL), and glutamine (2 mmol/L) (ThermoFisher, Waltham, MA, USA) in an incubator with 5% CO₂ at 37 °C (Isotemp Fisher Scientific, Pittsburgh, PA, USA), being subcultured every 2 days. The cells were seeded (5 × 10^5^ cells/well) and pre-incubated for 24 h. After that, the cells were treated with IBC and standard drug (chlorhexidine) at concentrations ranging from 25 to 0.39 μg/mL in 96 wells microplates for 24 h. Then, the culture medium was aspirated, and the cells were incubated with resazurin (70 μM, Sigma Aldrich) in the culture medium and re-incubated for another 4 h. Cell viability was read in a spectrophotometer (Biotek, Winooski, VT, USA) at wavelengths of 570 and 600 m. The values were converted into a percentage of cell viability in comparison with the negative control (DMEM), which was defined as having 100% cell metabolism. The means were determined for each compound [28].

## 3. Results and Discussion

### 3.1. Isobavachalcone (IBC)

**Scheme 1:**
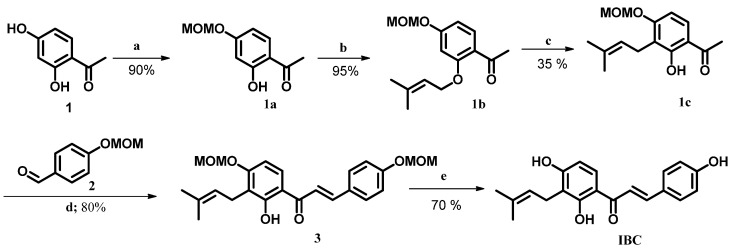
Synthesis of **IBC**. Reagents and conditions: (**a**) MOMCl, K_2_CO_3_, acetone, rt, 2 h; (**b**) isoprenyl bromide, K_2_CO_3_, acetone, rt, 24 h; (**c**) montmorillonite K10, DCM, rt, 0.5 h; (**d**) KOH 60%, EtOH, rt, 2 h; (**e**) HCl 1 mol L^−1^, MeOH:THF (1:1), 55 °C, 6 h. MOM = methoxymethyl.

Scheme 1 shows our synthetic route for synthesis of **IBC**. As illustrated, resacetophenone (**1**) was used as accessible starting material. A set of six steps, including MOM protection/desprotection, [1,3]-sigmatropic rearrangement and Claisen–Schmidt reactions furnished **IBC** with an overall yield of 12% (Scheme 1). NMR parameters, including chemical shifts, coupling constants and multiplicities as well as molecular weight corresponded to **IBC** structure and were compared with former literature reports [8,11]. HPLC-PAD analysis indicated 99% purity according to area peak at 372 nm.

### 3.2. Antibacterial and Antimycobacterial Activities

In order to assess the antibacterial and antimycobacterial activities, **IBC** was evaluated against five Gram-positive, two Gram-negative and three *Mycobacterium* species (Table 1). Among these species, five species are in the World Health Organization (WHO) priority list, justifying the discovery of innovative antibacterial agents [18]. **IBC** was active against *S. aureus, S. pneumoniae, S. sanguinis, S. sobrinus and S. mutans* planktonic cells, exhibiting MIC values ranging from 1.56 to 50.0 μg/mL. Among these, **IBC** displayed potent activity against MSSA and MRSA, demonstrating MIC values of 1.56 and 3.12 μg/mL. These data are according to studies on anti-*Staphylococcus aureus* effect of **IBC** against standard strains and clinical isolates [29,30,31,32]. **IBC** was inactive against *P. aeruginosa* and *K. pneumoniae* planktonic cells (MIC > 400 µg/mL).

Antimycobacterial assays indicated MIC values of 62.5 μg/mL against *M.* *avium*, *M. kansasii* and *M. tuberculosis*. Two studies described the antitubercular activity of **IBC** against *M. tuberculosis* [33,34]. In addition, **IBC** was described as an inhibitor of extracellular nuclease Rv0888 from *M. tuberculosis* H37Rv, which acts as a virulence factor and could be related to the persistence of *M. tuberculosis* in the host [35].

### 3.3. Checkerboard Assay

In anti-infective drug therapy, the association of drugs is a useful strategy to treat bacterial infections, enhancing the antibacterial potency and decreasing side effects [36,37]. In this context, hydroxychalcones and aminochalcones showed synergistic association with vancomycin [38,39]. Considering the potent activity of **IBC** against *S. aureus*, we used this species for the checkerboard assay. We evaluated the effect of the combination of **IBC** with vancomycin against MSSA and MRSA planktonic cells (Table 2). The association between **IBC** and vancomycin displayed FICI values of 2.5 and 2.0 against MSSA and MRSA, respectively, indicating an indifferent association.

### 3.4. Antibiofilm Assay

Biofilm can be described as a highly organized sessile community of cells, which are attached to a substratum, interface and complex polymeric matrix [40]. *S. aureus* has been known to infect and form a chronic biofilm infection in medical devices, being the most common pathogen associated with nosocomial infections [41,42]. Several chronic infections are associated with *S*. *aureus* biofilms, including osteomyelitis, periodontitis, chronic wound infections, chronic rhinosinusitis, endocarditis and ocular infections. After the attachment stage, *S. aureus* biofilms are difficult to eradicate with conventional antibacterial agents and the host response [41].

Current antibacterial drugs have not been effective against *S. aureus* biofilms, requiring its surgical removal [43]. Some therapeutic alternatives have been investigated, including antibacterial peptides, vaccines, matrix-degrading enzymes, modulation of quorum-sense system and small molecules inhibitors. Most of them are in preclinical development stage [41,43]. Xanthohumol, a prenylated chalcone from hop extracts, showed a potent anti-adherent and antibiofilm activities against *S*. *aureus* [44,45]. As part of our efforts in the discovery of novel antibiofilm agents, we described the effect of chalcones against bacterial and fungal biofilms [38,46,47].

**Figure 2:**
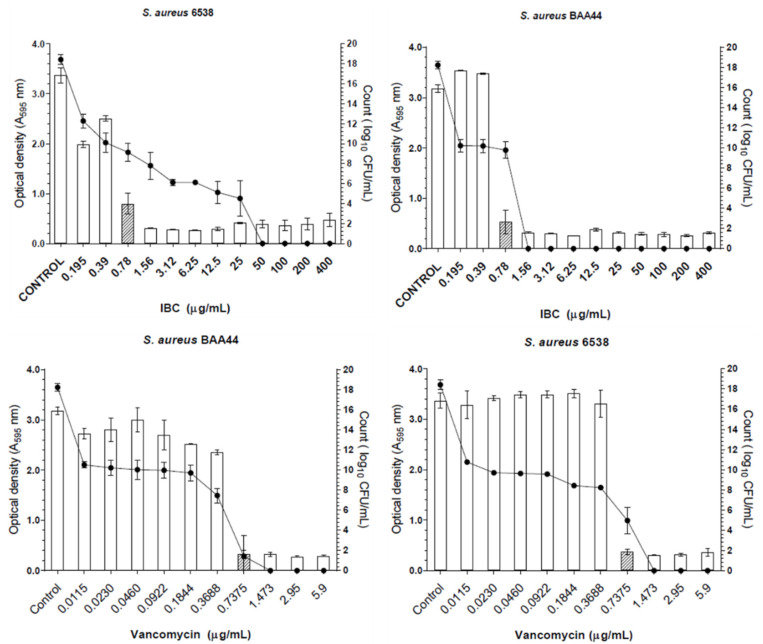
Effect of **IBC** and vancomycin on MSSA (*S. aureus* 6538) and MRSA (*S. aureus* BAA44) biofilm formation. The hatched bars represent the MBIC values. The lines represent the log_10_ CFU/mL.

**IBC** and vancomycin were evaluated in concentrations ranging from 0.195 to 400 µg/mL and 0.0115 to 5.9 µg/mL, respectively. **IBC** displayed antibiofilm activity with MBIC values of 0.78 µg/mL against MSSA and MRSA (Figure 2). At this concentration, **IBC** was able to inhibit 75% biofilm formation. Vancomycin displayed MBIC value of 0.74 µg/mL against MSSA and MRSA, demonstrating 90% biofilm formation inhibition. Moreover, in the presence of **IBC** at MBIC value, the number of viable cells of MSSA and MRSA decreased about 9 CFU/mL when compared with the untreated control.

### 3.5. Membrane Disruption Assay

**Figure 3:**
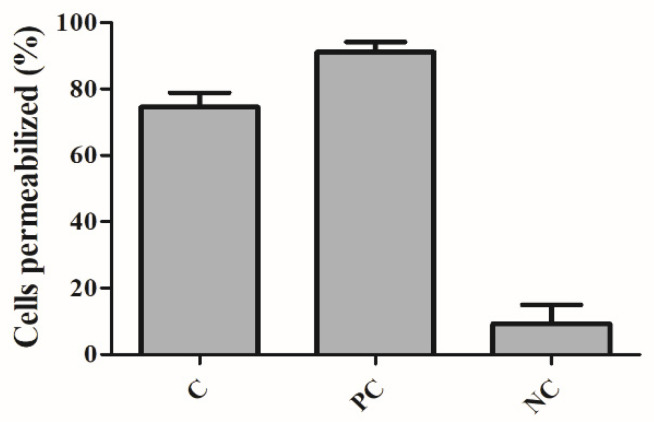
Percentage of disrupted *Bacillus subtilis* cells. C: cells treated with **IBC**; PC: cells treated with nisin (positive control) and NC: cells treated with 1% DMSO (negative control).

**Figure 4:**
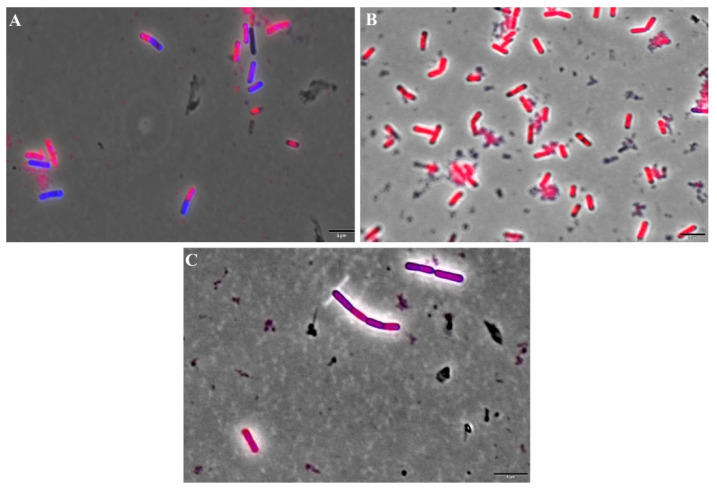
Fluorescence microscopy of *Bacillus subtilis* stained with propidium iodide and SYTO9. (**A**) negative control (cells treated with 1% DMSO); (**B**) positive control (cells treated with nisin at 5.0 µg/mL). (**C**) cells treated with **IBC**. Magnification 100×; Scale bar 5 μm.

In order to investigate the action of **IBC** on the bacterial membrane, we used *B. subtilis*, which is a non-pathogenic Gram-positive bacteria used for studies of antibacterial mode of action [26]. Bacterial cells were treated with **IBC** at its MBC value (3.13 µg/mL) for 15 min, as well as with nisin (positive control), an antibiotic that targets the bacterial membrane, producing pores [48]. Two fluorescent dyes propidium iodide (PI) and SYTO9 were added, which stain cells with disrupted membranes (in red) and intact membranes (in blue) [49]. Representative fluorescence microscopy images of the negative control (cells treated with 1% DMSO), the positive control (nisin at 5.0 µg/mL) and **IBC** were presented in Figure 3. The percentages of cells with damaged membranes were quantified from the microscope images (Figure 4). Cultures treated with 1% DMSO had approximately 10% of the cells with membranes permeabilized. Treatment with nisin demonstrated 95% of the cells stained with PI/SYTO9 due to its ability to makes pores in the bacterial membrane. Treatment of **IBC** displayed damage percentages of 75%.

Our finds are corroborated by other studies, which reported that the antibacterial mode of action of **IBC** is related to the disrupting action on bacterial membranes. **IBC** was able to cause damage to the MSSA membrane, evidenced by diS-C3-(5) dye experiments, leading to macromolecular biosynthesis inhibition [29]. Song and collaborators described how **IBC** binds to the phospholipids of MSSA membrane, resulting in the dissipation of proton motive force and metabolic perturbations [50]. Palko-Labuz and coauthors identified **IBC** as membrane-perturbing agent of human colorectal adenocarcinoma cells. **IBC** was able to be intercalated into model membranes, affecting phospholipid phase transition [51]. Additionally, the antibacterial effect of **IBC** has been correlated with the leakage of alkaline phosphatase (AKP) due to the impairment of the cell wall and cell membrane damage, the inhibition of protein and nucleic acids biosynthesis as well as the inhibition of energy metabolism [30,52].

### 3.6. Cytotoxicity Assay

**Figure 5:**
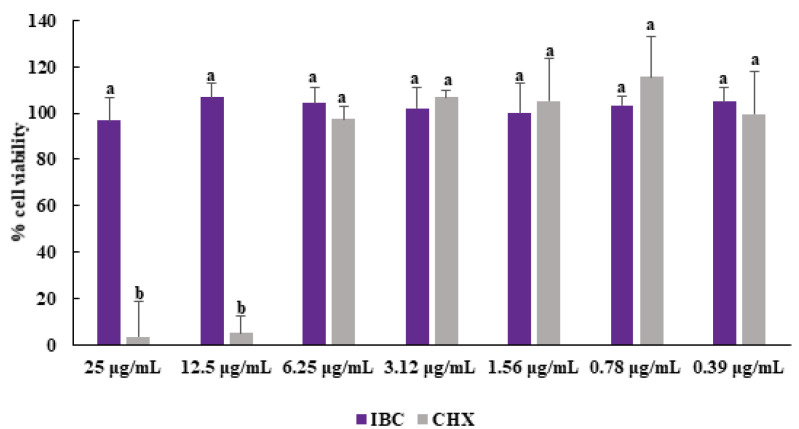
Effect of **IBC** and chlorhexidine on the viability of HaCaT cells. The results are expressed as means ± SDs. Different lowercase letters (a, b) show statistical differences between **IBC** and **CHX** according to ANOVA and Tukey’s test (*p* <0.05).

The evaluation of new antibacterial compounds against human cells is an important step for investigations of their selectivity and safety. The toxicity of **IBC** was tested against epidermal human keratinocytes (HaCaT cell line), which was chosen because skin is a typical site of *S. aureus* colonization [53]. **IBC** was not able to reduce the cell viability of HaCaT cells at the highest concentrations (25 µg/mL) after 24 h. (Figure 5). This concentration is approximatively 20 times higher than the MIC values obtained from assays MSSA and MRSA, indicating **IBC** has selectivity at its antibacterial concentration. Moreover, **IBC** demonstrated to be less cytotoxic than chlorhexidine at concentrations equal to or higher than 12.5 µg/mL.

In spite of the non-toxic effect of **IBC** against skin cells, several studies have reported its toxicity against leukemic cells [12] and solid tumor cells [54], including colorectal (HCT116) [55], tongue (Tca 8113) [56], liver (HepG2) [57], breast (MCF-7) [58], prostate (PC-3) [59], gastric (MGC803) [60], cervical (HeLa) [61], ovarian (OVCAR-08) [62] and neuroblastoma (IMR-32) [63]. The mechanism of **IBC** cytotoxicity is related to apoptosis induction via the mitochondrial pathway, decreasing its transmembrane potential [55,57,60,61,62,63].

## 4. Conclusions

In summary, we newly synthesized and evaluated **IBC** as part of our ongoing search for antibacterial agents. **IBC** has potent activity against Gram-positive (MIC = 1.56–50.0 µg/mL) and *Mycobacterium* species (MIC = 62.5 µg/mL). The combination of **IBC** and vancomycin exhibited indifferent effects against MSSA and MRSA planktonic cells. Antibiofilm activity of **IBC** was equipotent to vancomycin, displaying similar MBIC values. The mode of action of **IBC** involved membrane disruption, which is a crucial target for the bacterial survival. Furthermore, investigations of toxicity against human keratinocytes indicated that **IBC** is a selective compound. Altogether, our findings open new avenues for **IBC** as an antibacterial agent, with potential applications as a drug candidate and medical device coating.

## Figures and Tables

**Table 1:** Antibacterial and antimycobacterial activities of **IBC**.

	Species	MIC (µg/mL)			
		IBC	Tetracycline	Chlorhexidine	Isoniazid
Gram-positive	MSSA	1.56	0.20	-	-
	MRSA	3.12	>5.90	-	-
	*Streptococcus pneumoniae*	50.0	0.40	-	-
	*Streptococcus sanguinis*	3.12	-	1.82	-
	*Streptococcus sobrinus*	6.25	-	3.64	-
	*Streptococcus mutans*	6.25	-	0.91	-
	*Mycobacterium avium*	62.5	-	-	0.50
Mycobacteria	*Mycobacterium kansasii*	62.5	-	-	1.00
	*Mycobacterium tuberculosis*	62.5	-	-	>1.00
Gram-negative	*Pseudomonas aeruginosa*	>400	5.90	-	-
	*Klebsiella pneumoniae*	>400	2.95	-	-

MSSA = Methicillin-susceptible Staphylococcus aureus; MRSA = Methicillin-resistant Staphylococcus aureus.

**Table 2:** Antibacterial effect of combination of **IBC** and vancomycin against MSSA and MRSA.

*S. aureus* Strain	Combination	MIC (µg/mL)				FICI	Type of Combination
		Alone		Combined		FIC^IBC^ + FIC^VAN^	
		IBC	VAN	IBC	VAN		
MRSA	IBC + VAN	3.12	0.73	1.56	1.47	2.5	indifferent
MSSA	IBC + VAN	1.56	0.73	1.56	0.73	2.0	indifferent

VAN = vancomycin.

## Data Availability

Not applicable.

## Supplementary Materials

The following are available online at https://www.mdpi.com/article/10.3390/membranes12030269/s1, Figure S1: HPLC-PAD chromatogram of IBC. Methanol:Water (3:1), 372 nm, Figure S2. UV-Vis spectra of IBC, Figure S3. ^1^H NMR spectrum of IBC (acetone-*d*_6_; 600 MHz), Figure S4. ^13^C NMR spectrum of IBC (acetone-*d*_6_; 150 MHz), Figure S5. Mass spectrum (MS) of IBC (electrospray, positive mode).
