# Supplementary material for: Antibacterial Activity of Isobavachalcone (IBC) Is Associated with Membrane Disruption
Source: Membranes (Basel). 2022 Feb 25;12(3):269. doi: 10.3390/membranes12030269 (PMC8950343; doi:10.3390/membranes12030269)
Supplement: Supplementary file 1 [file membranes-12-00269-s001.zip › membranes-1474521-supplementary.pdf]

Antibacterial Activity of Isobavachalcone (IBC) is Associated with Membrane Disruption

Leticia Ribeiro de Assis[1], Reinaldo dos Santos Theodoro[1], Maria Beatriz Silva Costa[1], Julyanna Andrade Silva Nascentes[1], Miguel Divino da Rocha[1], Meliza Arantes de Souza Bessa[2], Ralciane de Paula Menezes[2], Guilherme Dilarri[3], Giovane Böerner Hypolito[3], Vanessa Rodrigues dos Santos[4], Cristiane Duque[4], Henrique Ferreira[3], Carlos Henrique Gomes Martins[2], Luis Octavio Regasini[1,*]

[1]Department of Chemistry and Environmental Sciences, Institute of Biosciences, Humanities and Exact Sciences, São Paulo State University (Unesp), São José do Rio Preto 15054-000, SP, Brazil.

[2]Department of Microbiology, Institute of Biomedical Sciences, Federal University of Uberlândia (UFU), Umuarama 38405-320, MG, Brazil.

[3]Department of Biochemistry and Microbiology, Institute of Biosciences, São Paulo State University (Unesp), Rio Claro 130506-900, SP, Brazil.

[4]Department of Preventive and Restorative Dentistry, School of Dentistry, São Paulo State University (Unesp), Araçatuba 16015-050, SP, Brazil

Correspondence: luis.regasini@unesp.br (LO Regasini)

SUPPLEMENTARY MATERIAL – Chemical Procedures

1. General Procedures

Melting point (mp) was measured using Melt Temperature apparatus MS Tecnopon PFM-II in open capillary tubes. NMR spectra were recorded on Bruker Avance III spectrometer, operating at 600 MHz for ^{1}H NMR and 150 MHz for ^{13}C NMR. Chemical shifts (δ) were referenced to residual non-deuterated solvent signals. Signal multiplicities were reported as singlet (s), and doublet (d). MS spectrum was recorded on Exactive Plus Thermo Scientific Electrospray Mass spectrometer, operating on positive mode. UV-Vis spectra and chromatograms were obtained by High Performance Liquid Chromatography (HPLC) with Photodiode Array Detector (HPLC-PAD) Agilent Technologies® 1220 Infinity LC equipment, photodiode array system (Agilent Tecnologies® Model 1260 Infinity) and Agilent Zorbax Eclipse Plus C-18® column (250 mm × 4.6 mm, 5 μm) using methanol:water (3:1) with 0.5% of acetic acid as mobile phase (1.0 mL/min). Purity of **IBC** was established through area of peak at 372 nm.

1.1.Synthesis

2’-hydroxy-4’-(methoxymethoxy)acetophenone (1a)

To a mixture of resacetophenone (3 mmol) and anhydrous K_2CO_3 (6 mmol) in acetone (10 mL). The methoxymethyl chloride (MOMCl) was added in portions for 30 min [1]. The mixture reaction was stirred for 2 h at room temperature. The reaction medium was extracted with ethyl acetate (3 × 25 mL) and combined organic phases were washed with brine, dried over $MgSO_4$, filtered and evaporated under reduced pressure to give **1a**, with 90% yield.

4-(methoxymethoxy)benzaldehyde (2)

To a mixture of 4-hydroxybenzaldehyde (3 mmol) and anhydrous K_2CO_3 (6 mmol) in acetone (10 mL). The methoxymethyl chloride (MOMCl) was added in portions for 30 min [1]. The mixture reaction was stirred for 5 h at room temperature. The reaction medium was extracted with ethyl acetate (3 × 25 mL), and the combined organic phases were washed with brine, dried over $MgSO_4$, filtered and evaporated under reduced pressure to furnish **2**, with 90% yield.

4'-(methoxymethoxy)-2'-prenyloxycetophenone (1b)

A mixture of **1a** (2.5 mmol) and K_2CO_3 (5 mmol) in acetone (5 mL) was stirred for 15 min at room temperature [1]. Isoprenyl bromide (3 mmol) was added in portions for 30 min and the reaction mixture was stirred for 24 h at room temperature. The reaction medium was extracted with ethyl acetate (3 × 25 mL) and the combined organic phases were washed with brine, dried over $MgSO_4$, filtered and evaporated under reduced pressure, to give 4-methoxymethoxy-2-*O*-prenyloxyacetophenone (**1b**) as a yellowish oil with 95% yield.

2'-hydroxy-4'-methoxymethoxy-3'-prenylacetophenone (1c)

To a stirred solution of **1b** (0.610g, 2.3 mmol) in dry dichloromethane (5 mL) was added montmorillonite K10 (0.610 g) at 0 ^{0}C and the reaction medium was stirred for 30 min at room temperature [1]. The reaction mixture was filtered and washed several times with ethyl acetate, which was reduced under vacuum, obtaining a brown oil. This residue was purified over silica gel column chromatography with hexane/ethyl acetate/acetone (9.5:0.25:0.25) to afford **1c** with 35% yield .

2'-hydroxy-4, 4'-Bis(methoxymethoxy)-3'-prenylchalcone (3)

To an ethanolic solution (3 mL) of **1b** (0.210 g, 0.8 mmol) was added dropwise under stirring an aqueous solution of KOH (60% w/v, 2 mL) at 0 ^{0}C [1]. 2-(methoxymethoxy)benzaldehyde (**2**, 0.166 g, 1 mmol) solubilized in EtOH (2 mL) was added to the mixture in portions for 1 h at room temperature. The reaction mixture was stirred for 2 h at room temperature and poured into crushed deionized ice and neutralized with HCl solution (1 mol/L). The mixture reaction was extracted with ethyl acetate (3 × 25 mL), and the organic phases were combined, washed with saturated aqueous $NaHSO_3$solution (2 ×10 mL) and brine, dried over $MgSO_4$, filtered, and concentrated under reduced pressure, furnishing chalcone **3** as a yellow oil, with 60% yield.

(*E*)-1-(2,4-dihydroxy-3-(3-methylbut-2-en-1-yl)phenyl)-3-(4-hydroxyphenyl)prop-2-en-1-one (IBC)

To a stirred solution of MOM-protected chalcone **3** (0.6 mmol, 0.264g) in 3 mL of MeOH:THF (1:1) was added 2 mL of HCl aqueous solution (1 mol/L) at 0 ^{0}C [1]. The reaction mixture was stirred at 55 ^{0}C for 6 h and poured into crushed deionized ice, filtered and the crude yellow powder was purified over silica gel column chromatography with hexane/ethyl acetate to afford **IBC**, with yield of 60%.

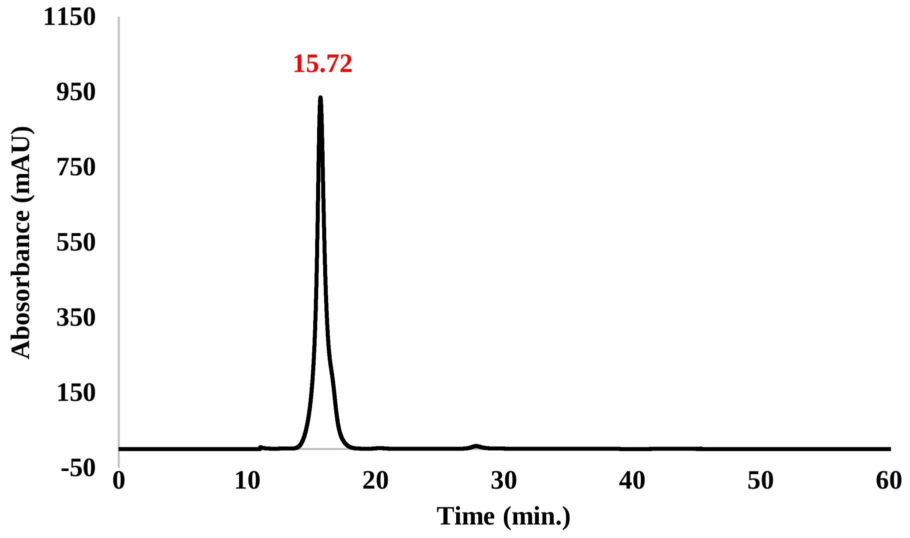

Figure S1. HPLC-PAD chromatogram of **IBC**. Methanol:Water (3:1), 372 nm

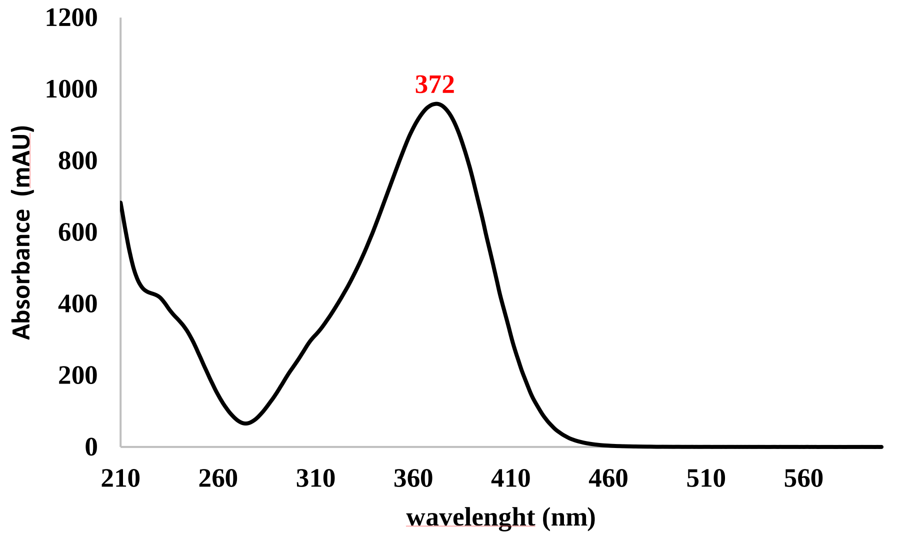

Figure S2. UV-Vis spectra of **IBC**

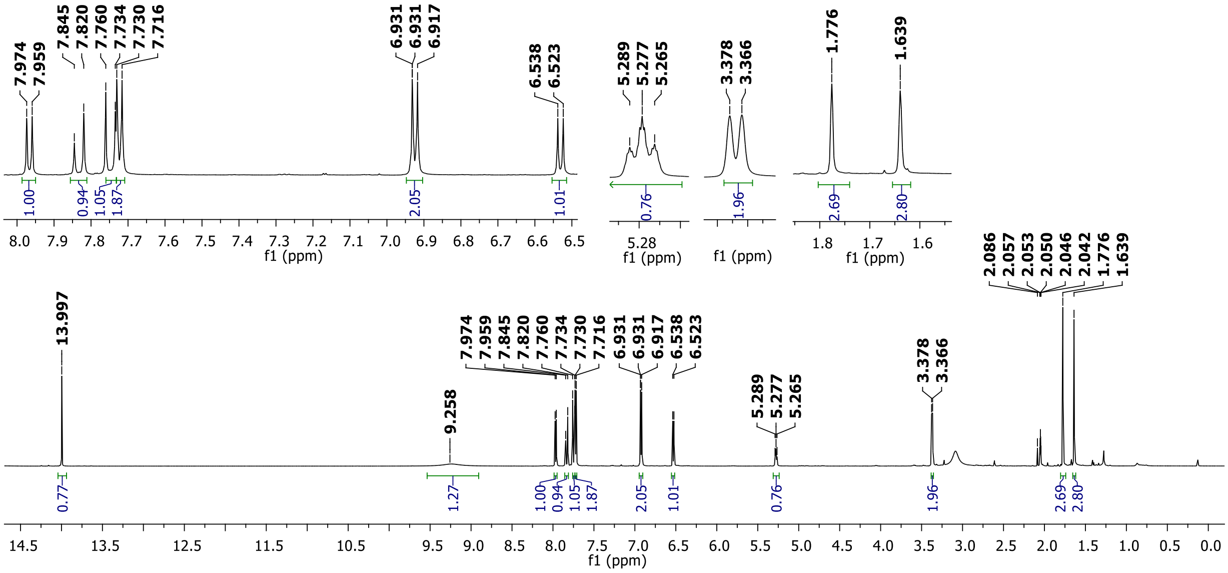

Figure S3. ^{1}H NMR spectrum of **IBC** (acetone-d_6; 600 MHz)

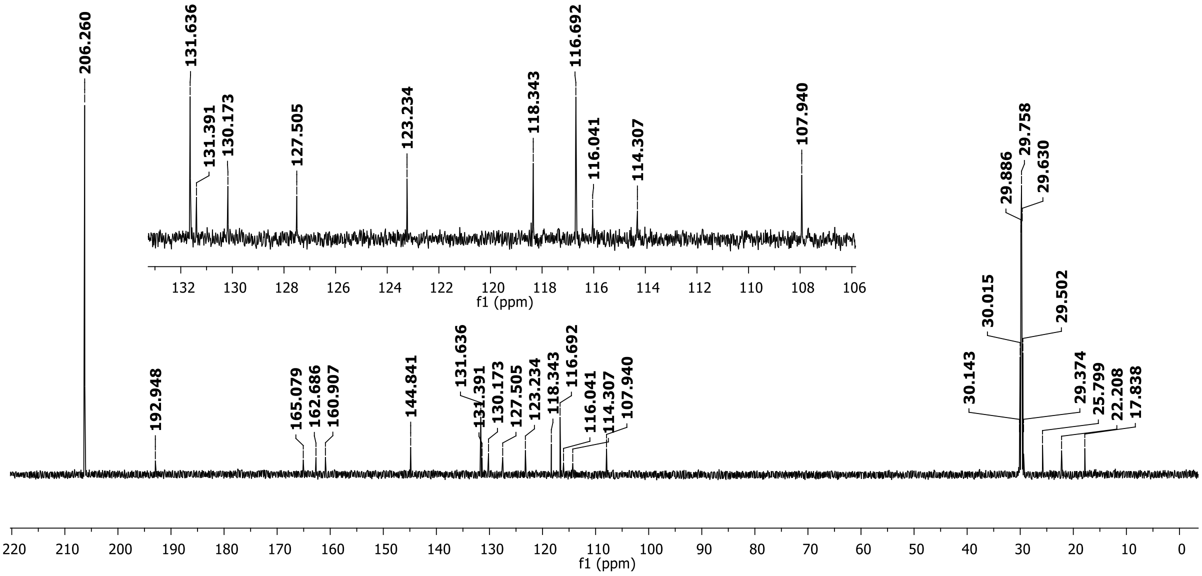

Figure S4. ^{13}C NMR spectrum of **IBC** (acetone-d_6; 150 MHz)

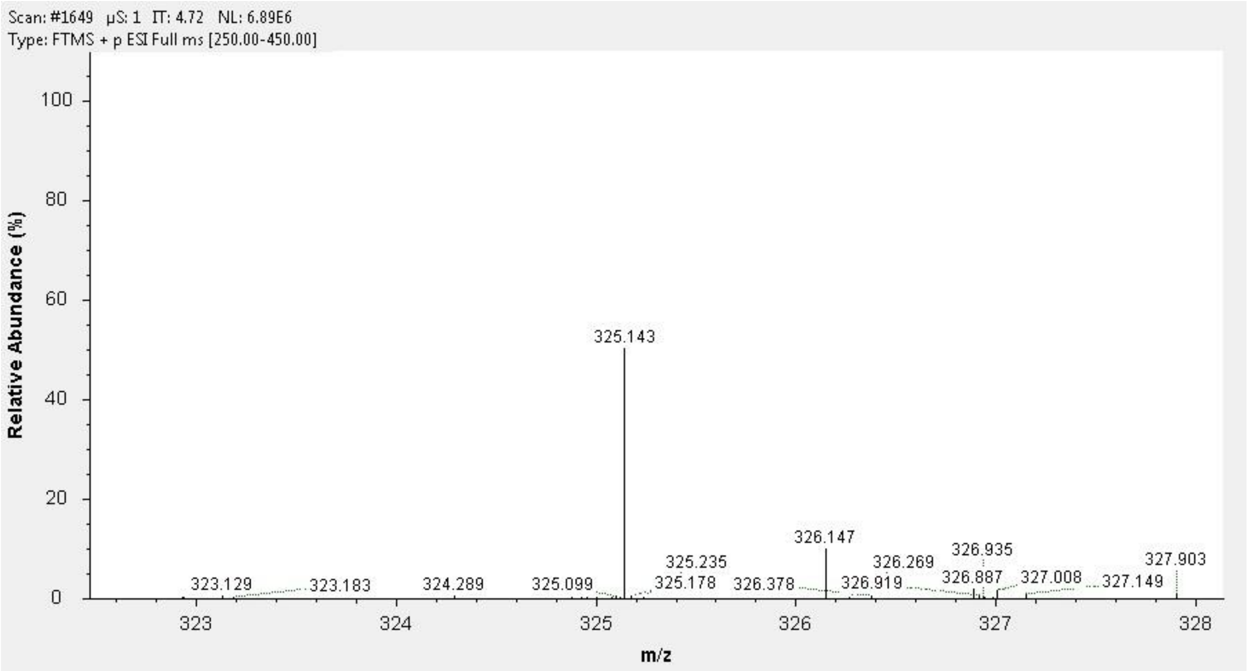

Figure S5. Mass spectrum (MS) of **IBC** (electrospray, positive mode)

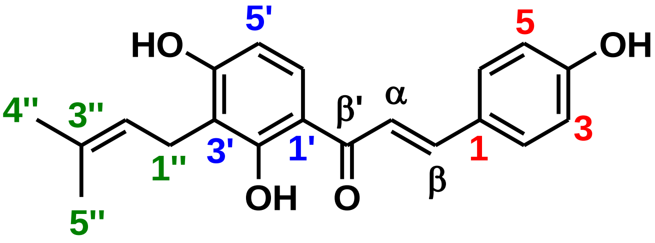

Yellow powder; HPLC purity: 99 %; m.p. literature: 156.8–157.8 ºC.[1], m.p. found: 134–137ºC. ^{1}H NMR (600 MHz, acetone-d_6) according to literature [2]: δ 1.64 (s, H-5”), 1.78 (s, H-4”), 3.37 (d, *J* = 7.2 Hz, H-1”), 5.28 (t, *J* = 7.2 Hz, H-2”), 6.53 (d, *J* = 8.8 Hz, H-5’), 6.92 (d, *J* = 8.6 Hz, H-3 and H-5), 7.72 (d, *J* = 8.6 Hz, H-2 and H-6), 7.75 (d, *J* = 15.4 Hz, H-α), 7.83 (d, *J* = 15.3 Hz, H-β), 7.97 (d, *J* = 8.9 Hz, H-6’), 9.26 (brs, 4’-OH), 14.0 (s, 2’-OH). ^{13}C NMR (150 MHz, acetone-d_6): δ 17.84 (C-5”), 22.20 (C-1”), 25.80 (C-4”), 107.9 (C-5’), 114.3 (C-1’), 116.0 (C-3’), 116.7 (C-3 and C-5), 118.3 (C-α), 123.2 (C-2”), 127.5 (C-1), 130.2 (C-6’), 131.4 (C-3”), 131.6 (C-2 and C-6), 144.8 (C-β), 160.9 (C-4), 162.7 (C-2’), 165.1 (C-4’), 192.9 (C-β’). UV/Vis λmax (MeOH): 372 nm. MS (Mwt: 324.136): *m/z* 325.143 (M+1) (base peak).

[1] Sugamoto K, Matsusita YI, Matsui K, Kurogi C, Matsui T. Synthesis and antibacterial activity of chalcones bearing prenyl or geranyl groups from Angelica keiskei. **Tetrahedron**; 2011;67:5346–59. doi: 10.1016/j.tet.2011.04.104

[2] Wang HM, Zhang L, Liu J, Yang ZL, Zhao HY, Yang Y, et al. Synthesis and anti-cancer activity evaluation of novel prenylated and geranylated chalcone natural products and their analogs. **Eur J Med Chem**.; 2015;92:439–48. doi: 10.1016/j.ejmech.2015.01.007
